# Gelam Honey Attenuates Carrageenan-Induced Rat Paw Inflammation via NF-κB Pathway

**DOI:** 10.1371/journal.pone.0072365

**Published:** 2013-08-28

**Authors:** Saba Zuhair Hussein, Kamaruddin Mohd Yusoff, Suzana Makpol, Yasmin Anum Mohd Yusof

**Affiliations:** 1 Department of Biochemistry, Faculty of Medicine, Universiti Kebangsaan Malaysia, Kuala Lumpur, Malaysia; 2 Department of Molecular Biology and Genetics, Faculty of Arts and Science, Canik Basari University, Samsun, Turkey; Universidade Federal do Rio de Janeiro, Brazil

## Abstract

The activation of nuclear factor kappa B (NF-κB) plays a major role in the pathogenesis of a number of inflammatory diseases. In this study, we investigated the anti-inflammatory mechanism of Gelam honey in inflammation induced rats via NF-κB signalling pathway. Rats paw edema was induced by subplantar injection of 1% carrageenan into the right hind paw. Rats were pre-treated with Gelam honey at different doses (1 or 2 g/kg, p.o.) and NSAID Indomethacin (10 mg/kg, p.o.), in two time points (1 and 7 days). Our results showed that Gelam honey at both concentrations suppressed the gene expressions of NF-κB (p65 & p50) and IκBα in inflamed rats paw tissues. In addition, Gelam honey inhibited the nuclear translocation and activation of NF-κB and decreased the cytosolic degradation of IκBα dose dependently in inflamed rats paw tissues. The immunohistochemical expressions of pro-inflammatory mediators COX-2 and TNF-α were also decreased in inflamed rats paw tissues when treated with Gelam honey. The results of our findings suggest that Gelam honey exhibits its inhibitory effects by attenuating NF-κB translocation to the nucleus and inhibiting IκBα degradation, with subsequent decrease of inflammatory mediators COX-2 and TNF-α.

## Introduction

Honey is a sweet and flavorful natural product of honey bees that is derived from floral nectars and other plant secretions [1]. The major component of honey is a complex mixture of sugars such as glucose, fructose and sucrose with small amount of other constituents including minerals, proteins, amino acids, enzymes, organic acids, vitamins, phenolic compounds [2]. For centuries, honey has been used for nutrition in different cultures and it has also been used as a traditional medicine due to its healing properties [3]. It has been reported to be effective in the treatment of gastrointestinal disorders [4], wounds and burns healing [5], asthma [6], cataracts [7], [8] and cancer [9]. Honey has also been shown to have antimicrobial, antiviral, antioxidant, anti-inflammatory and anticancer properties, in both *in vitro* and *in vivo* studies [10]–[14]. These properties are mainly attributed to the phenolic compounds in honey such as flavonoids which are recognized for their high pharmacological activities as antioxidant and radical scavengers [15], [16].

Inflammation is a complex biological response of the body against infections, irritations or other injuries, cell damage and vascularized tissues and is critical for both innate and adaptive immunity [17], [18]. Inflammation plays an important role in various diseases such as rheumatoid arthritis, asthma, inflammatory bowel disease, neurodegenerative diseases and cancer [19], [20]. During an inflammatory response, several pro-inflammatory mediators are released, including interleukin 6 (IL-6), IL-12, tumor necrosis factor (TNF), interferon (INF-γ), cyclooxygenase-2 (COX-2) and inducible nitric oxide synthase (iNOS) [20], [21]. These cytokines play major roles in the initiation and amplification of inflammatory processes [22]. Nuclear factor kappa B (NF-κB), transcription factor, also plays an important role in the inflammatory response by regulating the expression of various genes encoding pro-inflammatory mediators such as cytokines, chemokines, growth factors and inducible enzymes [23], [24].

NF-κB family consists of five proteins: NF-κB1 (p50/p105), NF-κB2 (p52/p100), RelA (p65), RelB and c-Rel [25]. It is found in the cytoplasm in an inactive form associated with regulatory proteins called inhibitors of κB (IκB) [26]. IκB kinase (IKK) complex is a key kinase which phosphorylates the protein IκB leading to proteasomal degradation of IκB and activation of the NF-κB [27]. Once activated, NF-κB is translocated to the nucleus from the cytoplasm, which then activates the genes related to inflammatory responses [28], [29]. Thus, inhibition of NF-κB could reduce the expression of inflammatory genes and is a mechanism by which anti-inflammatory agents might elicit their anti-inflammatory effects [30]. Various natural compounds have been shown to exhibit anti-inflammatory activity through inactivation of NF- κB via different mechanisms. For example, Gonzales and Orlando [31] reported that curcumin and resveratrol were able to inhibit TNF-α-activated NF-κB signaling in adipocytes and significantly reduced production of cytokines TNF-α, IL-1β, IL-6 and COX-2 genes expression. Yang et al. [32] found that green tea polyphenol epigallocatechin-3-gallate (EGCG) suppresses NF-κB activation by inhibiting IKK activity in intestinal epithelial cell line (IEC-6).

However, to date, no study has reported the elucidation of the mechanism by which Gelam honey exhibits its anti-inflammatory effect. Our previous study has shown that Gelam honey has anti-inflammatory effects by alleviating the rat paw edema and inhibiting the expression of pro-inflammatory mediators such as iNOS, COX-2, IL-6 and TNF-α in inflammation-induced paw edema in rats [33]. In the present study, we investigated further the anti-inflammatory effect of Gelam honey by elucidating its possible activation of NF-κB signaling pathway using acute inflammation rat model.

## Materials and Methods

### Chemicals

All chemicals and reagents used were of analytical grade. Indomethacin and Carrageenan were obtained from Sigma Chemicals Co. (USA). Trisma Base, dithiothreitol (DTT), sodium dodecyl sulfate (SDS), sodium chloride (NaCl), potassium chloride (KCl), magnesium chloride (MgCl_2_), Tween 20, phenyl methylsulfonyl fluoride (PMSF) and tetramethylethylenediamine (TEMED), all were supplied by Sigma (USA). HEPES (*N*-2-hydroxyethylpiperazine-*N*’-2-ethanesulfonic acid) was from PPA Laboratories (Austria), ethylenediaminetetraacitic acid (EDTA) from Calbiochem (USA), Triton X-100 from Gainland Chemical Company (UK), skim milk from Sunlac (Malaysia), formalin from Hopkins & Williams (England), glycerol from Merck, (Germany) and chemiluminescence was supplied by Perkin Elmer (USA). The materials ethyl alcohol and Xylene were obtained from BDH (England). The membrane polyvinylidene fluoride (PVDF) was purchased from GE Healthcare (USA).

### Honey Sample and Sterilization Process

Gelam honey is produced by *Apis mellifera* bees, and the nectar and pollen were collected by the bees from the plant *Melaleuca cajuputi* Powell, also known locally as the “Gelam tree”. It was purchased from the National Apiary, Department of Agriculture, Batu Pahat, Johor, Malaysia.

Gelam honey was packed in tight cap plastic bottles and placed in a box before sending to SINAGAMA, Malaysian Nuclear Agency for sterilization process. The sterilization process was conducted using cobalt-60 source (Model JS10000, Atomic Energy of Canada Ltd, Ontario, Canada). The box which contained Gelam honey was carried into a gamma-radiation chamber and circled the cobalt-60 source for 5 times to reach the dose of 25 kGy [34]. The irradiated Gelam honey was stored in the dark at room temperature until use.

### Animal Model and Experimental Design

Male Sprague-Dawley rats (n = 84) weighing (200–300) g, were obtained from the laboratory Animals Resource Unit, Faculty of Medicine, Universiti Kebangsaan Malaysia. The rats were housed in individual cages under standard conditions (temperature at 22±2°C for 12 hours light/12 hours dark cycle) with food (standard pellet diet) and water provided *ad libitum*. The rats were acclimatized for one week within the work area environment before the experiment begins. Each rat was used only once. The experimental protocols used in this study were approved by Animal Ethics Committee of Universiti Kebangsaan Malaysia (date of approval 17th March 2010: pp/BIOK.2010/Yasmin).

Carrageenan-induced paw edema model was used for the assessment of anti-inflammatory activity [35], as described previously in Hussein et al. [33]. Briefly, two models were employed in this study, with each model consisting of seven groups (n = 6 rats for each group). The first model represents rats that were pre-treated with Gelam honey for 1 day, while the second model represents rats that were pre-treated with Gelam honey for 7 days. In both of the models, rats were pre-treated orally with honey once daily at two different doses (1 and 2 g/kg of body weight). The negative control received an equivalent volume of vehicle (distilled water) and the positive control group received non steroidal anti-inflammatory drug (NSAID) Indomethacin (10 mg/kg of body weight) [36]. One hour after the last day of administration of Gelam honey, vehicle or Indomethacin, paw edema was induced by subplantar injection of 0.2 ml/paw of 1% freshly prepared carrageenan suspension in normal saline into the right hind paw of each rat in both models [37].

### Preparation of Tissue Samples for Western Blot and RT-PCR Analyses

Twenty-four hours after carrageenan injection, the rats were sacrificed by decapitation. Rats paw tissue segments were cut and washed in normal saline several times. The paw tissues were snap frozen in liquid nitrogen and stored at −80°C for Western blot and RT-PCR analyses.

### Primer Design and Quantitative RT-PCR

The primers sequence for rat NF-κB (p65 & p50), IκBα and housekeeping gene glyceraldehyde 3-phosphate dehydrogenase (GADPH) were designed from the sequence list of GeneBank database (National Centre for biotechnology Information, NCBI) and then blasted against GeneBank database sequences.

RT-PCR was performed for the detection of the mRNA expressions of NF-κB (p65 & p50) and IκBα. Total RNA was extracted from the tissue samples using an RNeasyMini kit (QIAGEN, USA) in an RNase-free environment, following the manufacturer’s protocol. The RNA amount and purity was measured using the NanoDrop 2000 spectrophotometer (Thermo Scientific, USA). RNA (1 µg) was reverse transcribed into single-stranded cDNA using an iScript cDNA synthesis kit (BIO-RAD, USA). Real-time PCR was carried out using SYBER Green detection (BIO-RAD, USA) in an automatic iQ5 thermocycler (BIORAD, USA). Master mixes of cDNA extract, nuclease-free H_2_O and SYBR Green solution were aliquot into each reaction tube which contained forward and reverse primers. The primer sequences for NF-κBp65 forward: 5′- TTCCCTGAAGTGGAGCTAGGA-3′ and reverse: 5′- CATGTCGAGGAAGACACTGGA -3′; for NF-κBp50 forward: 5′- AACGCATCCCAAGGTGCTGGAA-3′ and reverse: 5′- GCAGCTGGAAAAGCTCAAGCCA-3′; for IκBα forward: 5′- AAGGACGAGGATTACGAGCAG-3′ and reverse: 5′- CCCTTCACCTGACCAATCACT -3′; for GADPH forward: 5′- TCAAGAAGGTGGTGAAGCAG-3′ and reverse: 5′- AGGTGGAAGAATGGGAGTTG-3′. The cycling conditions were as follows: initial denaturation at 95°C for 3 minutes and amplification for 40 cycles (95°C for 10 seconds for the denaturation, 56°C for 30 seconds for the annealing and extension). The relative amount of gene expression, normalized to the internal control GAPDH, was calculated according to the following formula:
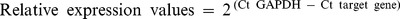



**Where:** Ct = The cycle at threshold level.

### Preparation of Cytosolic and Nuclear Protein Extracts

Protein extracts were prepared from paw tissues as described previously by Muthusamy et al. [38], with some modification. A 30 mg paw segment was ground with liquid nitrogen until it turned to powder form. An amount of 200 µl of a cytosolic/homogenizing buffer (10 mM HEPES, 10 mM KCL, 0.1 mM EDTA, 0.5 mM MgCl_2_, with freshly prepared 1 mM DTT, 0.1 mM PMSF and 1% Triton X-100, pH 7.9), was added and the homogenate was incubated in ice for 10 minutes. The homogenate was centrifuged at 5200 rpm for 5 minutes; the supernatant was collected and used as cytosolic protein extract. The nuclear pellet was collected and washed with the same volume of homogenizing buffer to remove cytosolic contaminants. Nuclear pellets were resuspended in a 100 µl of complete lysis buffer (20 mM HEPES, 420 mM NaCl, 0.1 mM EDTA, 1.5 mM MgCl_2_, 25% glycerol, 1 mM DTT and 0.5 mM PMSF, pH 7.9) and incubated at 4°C with mild shaking for 15 minutes. The supernatant was collected for nuclear protein extract after centrifugation at 8200 rpm for 5 minutes. The protein contents were determined using Bradford reagent [39].

### Western Blot Analysis

The tissue lysates (cytosolic or nuclear) containing equal amount of proteins (30 or 50 µg of total protein) were separated by 12% SDS-polyacrylamide gel and then transferred to *hybond-P (*PVDF) membrane. The membrane was blocked with 5% skim milk in TPBS solution (0.2% Tween 20 in PBS) for 1 hour. The membrane was then incubated overnight at 4°C with specific primary antibodies for the interest protein: NF-κBp65 (1∶500 dilution; Santa Cruz, USA), NF-κBp50 (1∶5000 dilution; Abcam, UK), IκBα (1∶10000 dilution; Abcam, UK), β-actin (1∶5000 dilution; Santa Cruz, USA) and Lamin B (1∶200 dilution; Santa Cruz, USA). The membrane was rinsed three times with TPBS solution for 5 minutes each. Thereafter, the membrane was incubated with secondary antibody (1∶3000) for 1 hour. The membrane was rinsed with TPBS solution for 5 minutes, which was repeated three times. Lastly, the membrane was incubated with 1 ml of chemiluminescence substrate for 5 minutes; the bands were visualized by Gel Documentation (Alpha Inno Tech, USA).

### Preparation of Tissue Samples for Histological Studies

The soft paw tissues were excised, fixed in 10% formalin for 24 hours and processed for paraffin embedding. Sections were cut at 3–4 µm thickness, flattened and adhered to the slides [40].

### Hematoxylin and Eosin (H&E) Staining and Inflammation Scoring

Paraffin-embedded sections (4 µm) were dewaxed by gradual washings in xylene followed by hydration with various concentrations of alcohol. H&E staining was performed on all samples for pathological examination. The stained sections were scored by two investigators in a blind fashion, and the degree of inflammation was evaluated according to Bang et al. [41] with a score from 0 to 5. The scores were defined as follows: 0 = no inflammation, 1 = mild inflammation, 2 = mild/moderate inflammation, 3 = moderate inflammation, 4 = moderate/severe inflammation and 5 = severe inflammation.

### Immunohistochemistry for Pro-inflammatory Mediators COX-2 and TNF-α

After dewaxing and dehydration, the sections were heated in target retrieval solution (TRS) for 20 minutes in a water bath at 98°C [high (pH = 9) for COX-2 and low (pH = 6) for TNF-α], for antigen recovery. After cooling at room temperature for 20 minutes, the sections were immersed in 3% hydrogen peroxide for 15 minutes at room temperature to abolish endogeneous peroxidase activities. The sections were incubated for 1 hour at room temperature with the primary antibodies (anti-COX-2 or anti- TNF-α, respectively) as 1∶1000 or 1∶400 dilutions in PBS-BSA. Then, the sections were washed in Tris buffer solution (TBS) and incubated for 30 minutes with secondary antibody (EnVisionTM FLEX kit; Dako, Denmark). After washing with TBS, the sections were stained with 3,3′diaminobenzidine-peroxide (DAB) chromophore, counterstained with hematoxylin, and mounted on microscope slides for analysis. Negative control was included by omitting the primary antibody, and positive controls for COX-2 and TNF-α were colon cancer tissues. The immunostaining for COX-2 and TNF-α were considered positive when the cytoplasm was stained brown. The cytoplasmic staining of COX-2 and TNF-α were evaluated by the percentage of positive cells according to the method of Habib et al. [42] and Lucetti et al. [40]. The mean percentage of positive staining cells was determined by counting 1000 stained cells at 10 different fields observed under 400X magnification using a light microscope [42].

### Statistical Analysis

All the data were presented as mean ± S.E.M. (n = 6). Statistical analysis was performed by one-way analysis of variance (ANOVA) using SPSS version 16.0 software. The statistical differences at level *p*<0.05 were considered significant.

## Results

### Effect of Gelam Honey on NF-κB (p65 & p50) and IκBα Gene Expressions in Paw Tissues

**Figure 1:**
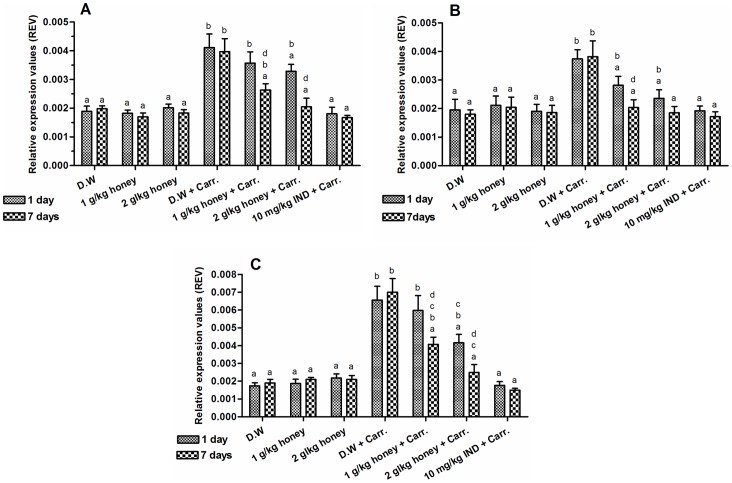
The effect of Gelam honey on the gene expressions in rats paw tissues. (**A**) NF-κB (p65) (**B**) NF-κB (p50) and (**C**) IκBα. Rats were pretreated orally with Gelam honey (1 or 2 g/kg of body weight) for 1 or 7 days before inflammation, as induced by carrageenan injection. D.W.: distilled water, Carr: Carrageenan, IND: Indomethacin. Data are presented as the mean ± S.E.M. (n = 6). **a**: Significantly different (*p*<0.05) from the inflammation group (D.W.+Carr.). **b**: Significantly different (*p*<0.05) from the Indomethacin group (10 mg/kg IND+Carr.). **c**: Significantly different (*p*<0.05) between different honey doses at the same time point (1 day or 7 days). **d**: Significantly different (*p*<0.05) between the same honey dose at different time points (1 day and 7 days).

The effect of Gelam honey on NF-κB (p65 & p50) and IκBα gene expressions in rats paw tissues were shown in **Figure 1**. The rats supplemented with Gelam honey at 1 and 2 g/kg of body weight, either 1 or 7 days, caused no significant change in p65, p50 and IκBα gene expressions compared with rats supplemented with distilled water (**Figures 1A–1C**). However, carrageenan injection led to significantly elevated levels of p65, p50 and IκBα gene expressions in the inflamed paws compared to control rats (not induced with inflammation). As shown from our results, the expression of p65, p50 and IκBα genes in inflamed rat paws were significantly (p<0.05) attenuated by the pre-treatment with Gelam honey (1 & 2 g/kg of body weight) in both 1 and 7 days models. Interestingly, the pre-treatment with 2 g/kg of body weight of Gelam honey for 7 days had greater effect on down-regulating the p65, p50 and IκBα gene expressions which was almost similar to the effect of the Indomethacin (10 mg/kg of body weight) (**Figures 1A–1C**).

### Effect of Gelam Honey on NF-κB (p65 & p50) Nuclear Translocation in Paw Tissues

**Figure 2:**
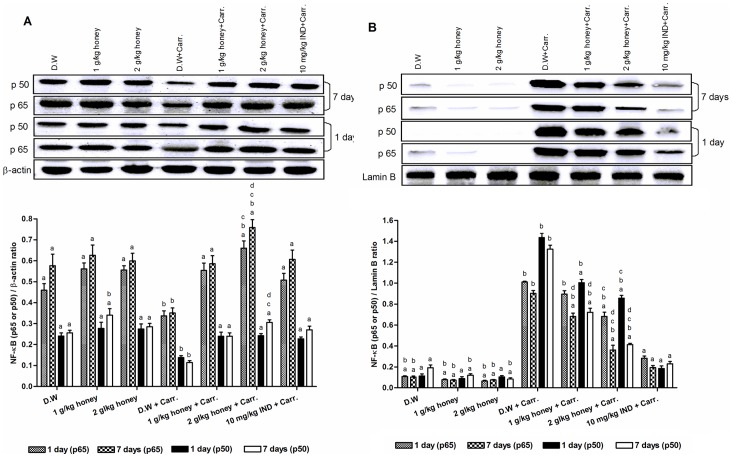
The effect of Gelam honey on the protein expressions in rats paw tissues. (**A**) Cytosolic NF-κB subunits p65 & p50 and (**B**) Nuclear NF-κB subunits p65 & p50. Rats were pretreated orally with Gelam honey (1 or 2 g/kg of body weight) for 1 or 7 days before inflammation, as induced by carrageenan injection. D.W.: distilled water, Carr: Carrageenan, IND: Indomethacin. Data are presented as the mean ± S.E.M. (n = 6). **a**: Significantly different (*p*<0.05) from the inflammation group (D.W.+Carr.). **b**: Significantly different (*p*<0.05) from the Indomethacin group (10 mg/kg IND+Carr.). **c**: Significantly different (*p*<0.05) between different honey doses at the same time point (1 day or 7 days) and same protein (p65 or p50). **d**: Significantly different (*p*<0.05) between the same honey dose at different time points (1 day and 7 days) and same protein (p65 or p50).

The results demonstrated that carrageenan injection induced the nuclear translocation of NF-κB subunits p65 and p50, as shown in **Figures 2A & 2B**. However, the cytoplasmic level of p65 and p50 increased significantly by the pre-treatment with Gelam honey (1 & 2 g/kg of body weight) in both 1 and 7 days models (**Figure 2A**). Simultaneously, the nuclear levels of p65 and p50 decreased significantly with Gelam honey pre-treatment (1 & 2 g/kg of body weight) for 1 and 7 days (**Figure 2B**), with concomitant increased of their cytoplasmic levels (**Figure 2A**). Pre-treatment with 2 g/kg of body weight of Gelam honey for 7 days showed greater effect in decreasing the nuclear p65 and p50 levels in parallel with their increased cytosolic levels.

### Effect of Gelam Honey on Cytoplasmic Degradation of IκBα

**Figure 3:**
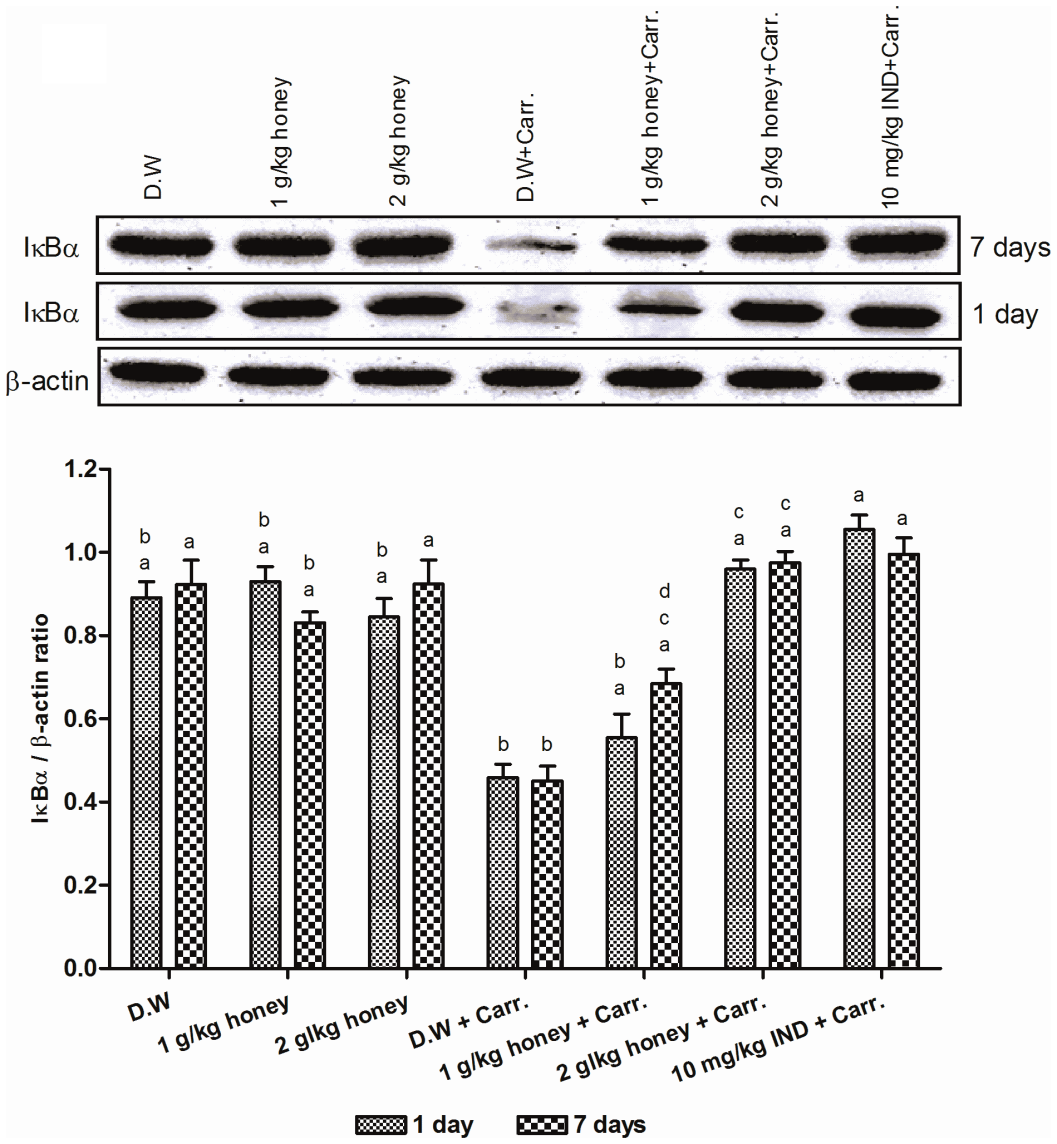
The effect of Gelam honey on the cytosolic IκBα protein expression in rats paw tissues. Rats were pretreated orally with Gelam honey (1 or 2 g/kg of body weight) for 1 or 7 days before inflammation, as induced by carrageenan injection. D.W.: distilled water, Carr: Carrageenan, IND: Indomethacin. Data are presented as the mean ± S.E.M. (n = 6). **a**: Significantly different (*p*<0.05) from the inflammation group (D.W.+Carr.). **b**: Significantly different (*p*<0.05) from the Indomethacin group (10 mg/kg IND+Carr.). **c**: Significantly different (*p*<0.05) between different honey doses at the same time point (1 day or 7 days). **d**: Significantly different (*p*<0.05) between the same honey dose at different time points (1 day and 7 days).

**Figure 3** showed that carrageenan injection induced the cytoplasmic degradation of IκBα protein showing low levels of IκBα when compared with the control groups (distilled water and two doses of Gelam honey) not induced with inflammation which exhibited high level of cytoplasmic IκBα protein. However, the cytoplasmic level of IκBα increased significantly by the pre-treatment with Gelam honey (1 and 2 g/kg of body weight) in both 1 and 7 days models. Interestingly, pre-treatment with 2 g/kg of body weight of Gelam honey inhibited the IκBα cytoplasmic degradation dose dependently showing more levels of cytosolic IκBα compared to the effect of lower dose of Gelam honey (1 g/kg of body weight) in both 1 and 7 days models (**Figure 3**).

### Histological Analyses of Rats Paw Tissues

**Figure 4:**
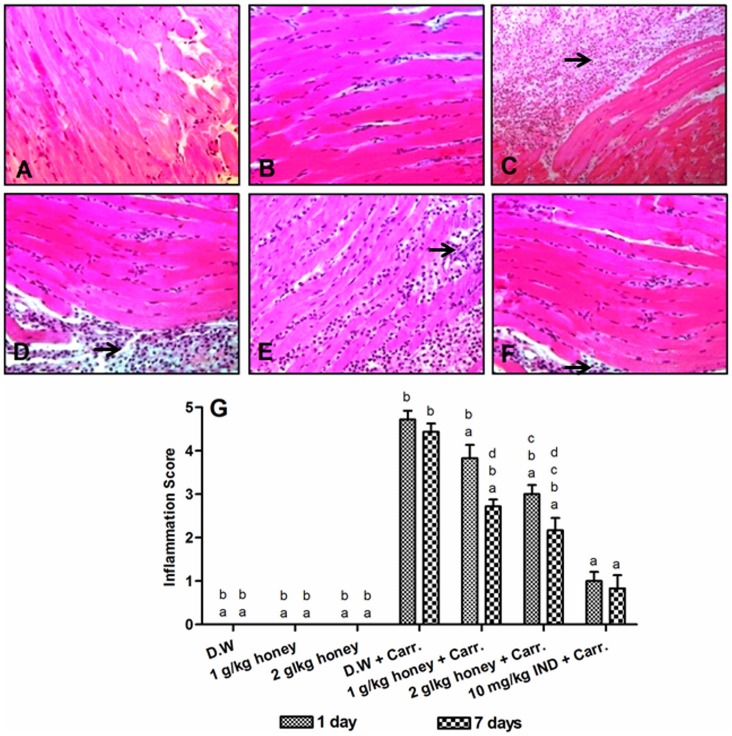
Histological evaluation of anti-inflammatory effects of Gelam honey. Hematoxylin and Eosin staining of paw tissues of rats pre-treated with: (**A**) D.W., (**B**) 2 g/kg Gelam honey, (**C**) D.W.+Carr., (**D**) 2 g/kg Gelam honey for 1 day+Carr. (**E**) 2 g/kg Gelam honey for 7 days+Carr. and (**F**) 10 mg/kg IND.+Carr., in the model of carrageenan-induced edema. Each photo is representative of six specimens for each group. All figures were magnified by 200X. (**G**) Scores of inflammation in rats paw tissues by Hematoxylin and Eosin staining. Data are expressed as mean ± S.E.M. (n = 6). D.W: Distilled water, Carr.: Carrageenan and IND: Indomethacin. **a**: Significantly different (*p*<0.05) from the inflammation group (D.W.+Carr.). **b**: Significantly different (*p*<0.05) from the Indomethacin group (10 mg/kg IND+Carr.). **c**: Significantly different (*p*<0.05) between different honey doses at the same time point (1 day or 7 days). **d**: Significantly different (*p*<0.05) between the same honey dose at different time points (1 day and 7 days). The arrows indicated inflammatory cells.

To evaluate histologically the anti-inflammatory effect of Gelam honey, samples of the paw tissues from each experimental group were examined by H&E staining. The control groups which were fed with distilled water and Gelam honey at 1 or 2 g/kg of body weight (not induced with inflammation) showed normal paw tissue histology (**Figures 4A & 4B**). In contrast, the carrageenan injection into the rat right hind paw displayed massive accumulation of infiltrated inflammatory cells (**Figure 4C**), compared to the control groups. However, the infiltration of inflammatory cells was significantly decreased with treatment of Gelam honey (1 or 2 g/kg of body weight) or Indomethacin (10 mg/kg of body weight), for both 1 and 7 days model (**Figures 4D–4F**). The degree of inflammation was evaluated by scores of inflammation from 0 to 5 (**Figure 4G**). The scores of inflammation also indicated that pre-treatment with Gelam honey (1 & 2 g/kg of body weight) for 7 days had greater effect in reducing the inflammatory cells in carrageenan-induced inflammation rats compared to 1 day.

### Immunohistochemistry Detection of COX-2 Protein Expression

**Figure 5:**
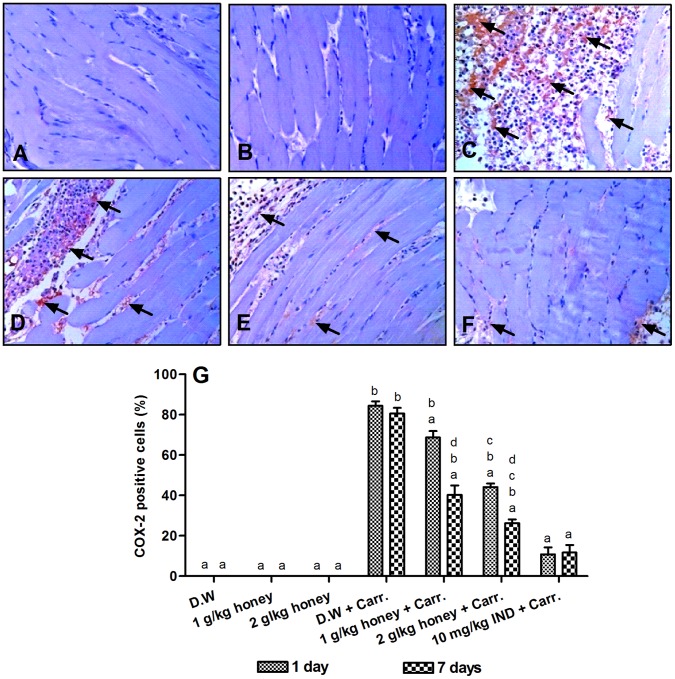
Immunohistochemical staining for COX-2 expression in the paw tissues. Rats were pre-treated with: (**A**) D.W., (**B**) 2 g/kg Gelam honey, (**C**) D.W.+Carr., (**D**) 2 g/kg Gelam honey for 1 day+Carr., (**E**) 2 g/kg Gelam honey for 7 days+Carr. and (**F**) 10 mg/kg IND.+Carr. All figures were magnified by 200X. The arrows indicated positive staining of COX-2 (400X). (**G**) Comparison of the percentage of cells stained with COX-2 in rats paw tissues. Data are expressed as mean ± S.E.M. (n = 6). D.W: Distilled water, Carr.: Carrageenan and IND: Indomethacin. **a**: Significantly different (*p*<0.05) from the inflammation group (D.W.+Carr.). **b**: Significantly different (*p*<0.05) from the Indomethacin group (10 mg/kg IND+Carr.). **c**: Significantly different (*p*<0.05) between different honey doses at the same time point (1 day or 7 days). **d**: Significantly different (*p*<0.05) between the same honey dose at different time points (1 day and 7 days).

To further elucidate the mechanism of action of Gelam honey, the protein expressions of some pro-inflammatory mediators such as COX-2 and TNF-α was determined using immunohistochemistry technique. As shown in **Figures 5A & 5B**, the normal control groups showed no expression of COX-2. However, the carrageenan-induced inflammation group showed significantly increased expression of COX-2 (80.6%) (**Figure 5C**), which was reduced significantly with pre-treatment of Gelam honey (1 or 2 g/kg of body weight) or Indomethacin (10 mg/kg of body weight), for 1 and 7 days (**Figures 5D–5F**). As expected, pre-treatment with 2 g/kg of body weight of Gelam honey for 7 days showed significantly higher reduction effect of COX-2 expression (26.29%) when compared with 1 day (44.14%) (**Figure 5G**).

### Immunohistochemistry Detection of TNF-α Protein Expression

**Figure 6:**
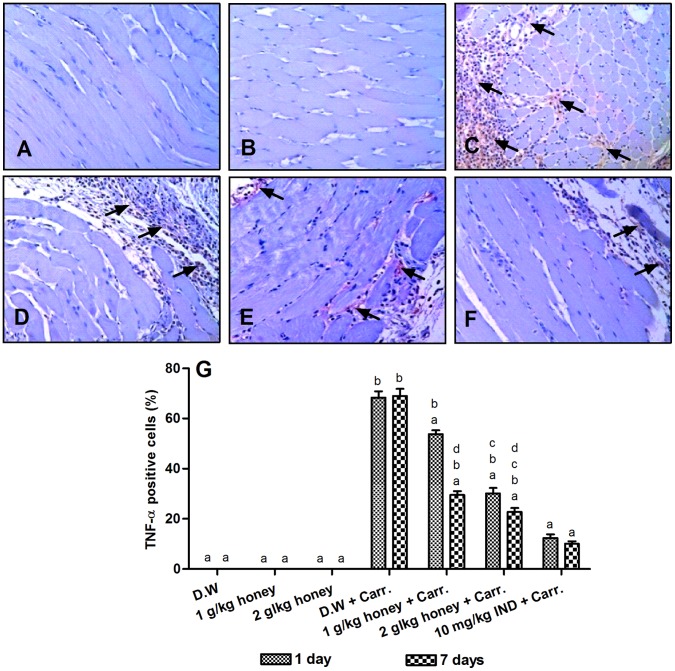
Immunohistochemical staining for TNF-α expression in the paw tissues. Rats pre-treated with: (**A**) D.W., (**B**) 2 g/kg Gelam honey, (**C**) D.W.+Carr., (**D**) 2 g/kg Gelam honey for 1 day+Carr. (**E**) 2 g/kg Gelam honey for 7 days+Carr. and (**F**) 10 mg/kg IND.+Carr. All figures were magnified by 200X. The arrows indicated positive staining of TNF-α (400X). (**G**) Comparison of the percentage of cells stained with TNF-α in rats paw tissues. Data are expressed as mean ± S.E.M. (n = 6). D.W: Distilled water, Carr.: Carrageenan and IND: Indomethacin. **a**: Significantly different (*p*<0.05) from the inflammation group (D.W.+Carr.). **b**: Significantly different (*p*<0.05) from the Indomethacin group (10 mg/kg IND+Carr.). **c**: Significantly different (*p*<0.05) between different honey doses at the same time point (1 day or 7 days). **d**: Significantly different (*p*<0.05) between the same honey dose at different time points (1 day and 7 days).

The expression of TNF-α in the carrageenan-induced inflammation group was significantly increased compared to the control group (68.95%), but pre-treatment with Gelam honey for 1 and 7 days (1 or 2 g/kg of body weight) reduced its expression comparably with Indomethacin (10 mg/kg of body weight), as shown in **Figures 6A–6F**. Pre-treatment for 7 days with Gelam honey either 1 or 2 g/kg of body weight significantly reduced the TNF-α expression (29.53% and 22.80%, respectively), when compared with 1 day pre-treatment (53.75% and 30.11%, respectively) (**Figure 6G**).

## Discussion

Inflammation is part of host defence mechanism against infectious agents and injury and it is also involved in the pathophysiology of many diseases when left unresolved. Acute inflammation is a short-term process which is characterized by typical signs of inflammation, such as swelling, heat, redness, pain and loss of function due to the infiltration of the tissues by plasma and leukocytes [43], [44]. Several pro-inflammatory mediators were released during the inflammatory response including iNOS, COX-2, TNF-α and IL-6 and the induction of these pro-inflammatory mediators are largely regulated by transcriptional activation [45].

NF-κB is known to be a major transcription factor that regulates the genes expression of pro-inflammatory mediators which participate in the inflammatory response [46], [47]. NF-κB proteins, p65 and p50, exist normally in the cytoplasm as an inactive complex by binding to inhibitory factor, IκBα, thereby blocking NF-κB nuclear translocation. Upon stimulation with inflammatory stimuli, IκBα is phosphorylated by IκB kinase (IKK) and separated from the NF-κB subunits which lead to its degradation. The free NF-κB is translocated into the nucleus and acts as transcription factor. In the nucleus, NF-κB dimers combine with target DNA elements to activate transcription of genes encoding for proteins involved in inflammation [48], [49]. In inflammation, activated NF-κB regulates transcription of IL-1β, IL-6, iNOS, COX-2 and TNF-α [50]. In order to reduce inflammation, the major focus has been on inhibiting NF-κB activation and its translocation into the nucleus thereby decreasing the production of pro-inflammatory mediators. Several natural products such as green tea, curcumin, ginger and propolis have been shown to ameliorate inflammatory response by inhibition of the pro-inflammatory mediators such as iNOS, COX-2 and TNF-α as well as inactivation of the NF-κB [51]–[53]. Our previous study on the anti-inflammatory properties of Gelam honey showed that it reduces edema in a dose dependent fashion in inflamed rat paw with concomitant reduced production of pro-inflammatory mediators (both genes and proteins) such as iNOS, COX-2, IL-6 and TNF-α in plasma and tissues [33]. In addition, we have also reported that Gelam honey contains many phenolic compounds such as ellagic acid, gallic acid, caffeic acid, quercetin and chrysin, which correlated to its antioxidant and anti-inflammatory activities [16], [33].

In this study we evaluated the mechanism by which Gelam honey exerts its anti-inflammatory effect in association with NF-κB signaling pathway by evaluating the genes and proteins that are involved in the pathway. The findings of this study confirmed the nuclear translocation of NF-κB subunits (p65 and p50) and cytoplasmic degradation of IκBα during inflammation as induced in rats by carrageenan injection. However, pre-treatment with Gelam honey significantly inhibited the nuclear translocation of NF-κB subunits (p65 and p50) as supported by increased levels of p65 and p50 protein in the cytosol and decreased cytosolic IκBα degradation. Various *in vitro* and *in vivo* experimental models have reported the inhibitory effects of several natural products and polyphenols on the production of pro-inflammatory mediators through attenuating the activation of NF-κB pathway. Oh et al. [27] reported that *Euonymus alatus* extract, a known medical herb in Korea, significantly attenuated the LPS-induced IκBα phosphorylation/degradation, NF-κB translocation from cytoplasm to the nucleus and subsequent NO synthesis in RAW 264.7 cells. Garcia-Mediavilla et al. [54] reported that quercetin significantly inhibited protein and mRNA levels of iNOS and COX-2 in the human hepatocyte-derived Chang liver cells. In animal study, Ukil et al. [55] found that curcumin reduced the levels of NO and iNOS expression associated with suppression of NF-κB activation in trinitrobenzene sulphonic acid (TNBS)-induced colitis in mouse. Zhang et al. [56] demonstrated that purple sweet potato inhibits the up-regulation of the expression of p65 NF-κB associated with the reduction of COX-2 and iNOS expressions in _D_-galactose-induced liver injury in mouse. Moreover, Hsu et al. [53] found that the combination of soy and tea inhibited nuclear NF-κB p50 protein level via induction of IκBα that lead to decreased TNF-α and IL-6 proteins expression in hormone-induced prostate cancer in rats. Therefore, our results strongly suggest that a possible mechanism of Gelam honey-mediated anti-inflammatory activity could be regulated by the action of phenolic compounds such as gallic acid, caffeic acid, hesperetin and quercetin which were found as the active principle of Gelam honey [16]. Polyphenols in honey have been shown to be bioavailable by Schramm et al [57] who reported that consumption of 1.5 g of two types of honey/kg body weight among 40 subjects increased plasma level of total phenolic content similar to antioxidant and reducing capacity of plasma [57]. In another study by Alvarez-Suarez et al, both Cuban honey extract and the flavonoid quercetin were shown to protect erythrocytes membrane against oxidative damage. Quercetin was found to be incorporated into the cell membrane. [58]. Phenolic aglycons have been proposed to be the major form of flavonoid in honey and it is more readily absorbed through the gut barrier than their corresponding glycosides by passive diffusion, thus making flavonoids in honey to be more readily bioavailable. [59]. In a separate study by Batumalei et al [60] it was shown that pretreatment of hamster pancreatic cells in culture (HIT-T15 cells) with Gelam honey extract (Melaleuca spp.) or the flavonoid components (chrysin, luteolin, and quercetin), prior to stimulation by 20 or 50 mM glucose showed a significant decrease in the production of ROS, glucose-induced lipid peroxidation, and a significant increase in insulin content and the viability of cells cultured under hyperglycemic condition showing the bioavailability of flavonoid in honey offering protection to pancreatic beta cells from oxidative damage.

Histological study indicated that carrageenan-induced inflammation is linked to intense edema characterized by increased migration of infiltrates inflammatory polymorphonuclear leukocytes (PMNs) cells, mainly neutrophils, in the inflamed paw tissues. Our results are consistent with other carrageenan-induced inflammation animal model showing increased neutrophils migration to the site of injury [40], [61]–[63]. Interestingly, Gelam honey treatment drastically and dose-dependently diminished the inflammatory cell migration, most likely neutrophils, in carrageenan-induced rat paw edema. We also observed that the effect was similar to Indomethacin, a well known NSAID anti-inflammatory drug. Bang et al. [41] reported that piperine, a phenolic compound in black pepper extract, significantly reduced the inflammatory cells infiltration in ankle joints of carrageenan-induced arthritis rats. Lucetti et al. [40] also found that lupeol acetate, which was isolated from *Himatanthus drasticus* Plumel (medicinal plant in Northeast Brazil), led to significant decrease in inflammatory cells infiltration of carrageenan-induced paw edema in mouse. Sindhu et al. [64] showed that treatment with *Trigonella foenum graecum*, an herbaceous plant of the leguminous family, significantly decreased the neutrophil infiltration in inflamed paws of adjuvant induced arthritis in rats. Thus, in line with other studies, the present histological results revealed that severe edema formation and elevated level of cellular infiltration in rats paw tissues decreased when treated with Gelam honey, in a dose dependent manner.

In order to validate the protein expression, histological expression of two proteins, namely COX-2 and TNF-α were determined. The reason of choosing these two proteins is that COX-2 is an important key enzyme in inflammation and it is also the rate-limiting enzyme that catalyzes the formation of prostaglandins. TNF-α is a pro-inflammatory cytokine which plays several important roles in inflammation based on its appearance at the inflammatory site and ability to induce certain mechanisms including activation and chemotaxis of leukocytes, expression of adhesion molecules on neutrophils and endothelial cells, and regulation of the secretion of other pro-inflammatory cytokines [65]–[67]. Our immunohistochemistry results showed that the expressions of COX-2 and TNF-α were significantly increased in rats paw tissues after carrageenan-induced inflammation when compared with control rats (without inflammation) which is in agreement with other studies showing increased expressions of COX-2 and TNF-α in the paw tissues of mouse and rats submitted to carrageenan-induced inflammation [40], [63], [68]. We showed that pre-treatment with Gelam honey significantly reduced the expressions of COX-2 and TNF-α dose-dependently in rats paw tissues showing similar reduction effect with Indomethacin. Cui et al. [69] found that resveratrol reduced iNOS, COX-2 and TNF-α expressions in dextran sulphate sodium (DSS)-induced colitis in mouse. In addition, ginger extract (*Zingiber officinale*) significantly reduced the elevated expression of NF-κB and TNF-α in ethionine-induced hepatoma rats [42].

**Figure 7:**
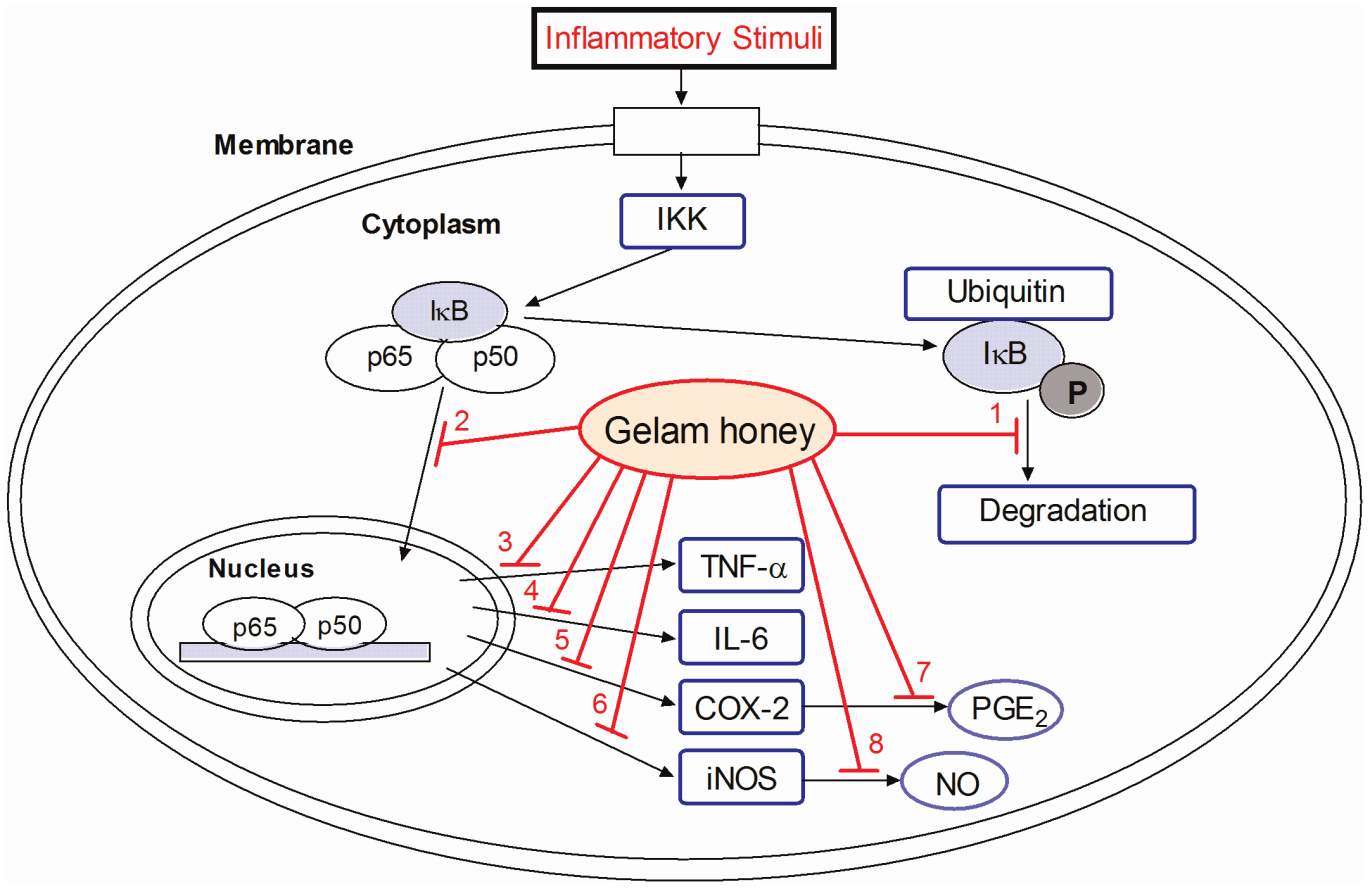
Proposed mechanism of action by which Gelam honey inhibited inflammation in carrageenan-induced rats paw edema. Activation of NF-κB pathway by different inflammatory stimuli leads to phosphorylation of inhibitor of kappa B (IκBα) by IκB kinase (IKK) which separates the NF-κB dimers (p50 and p65) and leads to the degradation of IκBα. The free NF-κB translocates from the cytoplasm into the nucleus and acts as transcription factor. In the nucleus, NF-κB dimers combine with target DNA elements to activate transcription of genes encoding for proteins involved in inflammation such as cyclooxygenase (COX-2), inducible nitric oxide synthase (iNOS), tumor necrosis factor-α (TNF-α) and interleukin-6 (IL-6). The study indicated that Gelam honey inhibited **(1)** the degradation of IκBα and leading to **(2)** nuclear translocation of NF-κB dimer (p50 and p65) resulting in the decreased expressions of **(3)** TNF-α, **(4)** IL-6, **(5)** COX-2 and **(6)** iNOS, as well as reduced production of **(7)** prostaglandin E2 (PGE_2_) and **(8)** nitric oxide (NO).

Our proposed mechanism by which Gelam honey inhibits inflammation in carrageenan-induced paw edema in rats is depicted in **Figure 7**. Gelam honey may inhibit inflammation through suppression of NF-κB pathway by blocking the incoming signal pathway which activates the IKK complex, thus interfering in the phosphorylation, ubiquitination and degradation of IκB proteins. This will then prevent the translocation of NF-κB dimers (p65 and p50) into the nucleus finally resulting in the reduction of iNOS, COX-2, TNF-α and IL-6 expressions as well as PGE_2_ and NO production, as shown in our previous study [33].

## Conclusion

In conclusion, our results strongly suggest that Gelam honey inhibited inflammation via inactivation of NF-κB, blocking IκBα degradation and nuclear translocation of p65 and p50 NF-κB subunits thereby inhibiting the binding of NF-κB to its target DNA resulting in inhibition of transcription of genes for pro-inflammatory mediators such as COX-2, TNF-α, IL-6 and iNOS. Interestingly, the results of this study indicated that NF-κB is a pivotal transcription factor for inflammatory signal pathway.
